# High Levels of Adherence and Viral Suppression in a Nationally Representative Sample of HIV-Infected Adults on Antiretroviral Therapy for 6, 12 and 18 Months in Rwanda

**DOI:** 10.1371/journal.pone.0053586

**Published:** 2013-01-09

**Authors:** Batya Elul, Paulin Basinga, Harriet Nuwagaba-Biribonwoha, Suzue Saito, Deborah Horowitz, Denis Nash, Jules Mugabo, Veronicah Mugisha, Etienne Rugigana, Richard Nkunda, Anita Asiimwe

**Affiliations:** 1 ICAP at Columbia University, Mailman School of Public Health, New York, New York, United States of America; 2 Department of Epidemiology, Mailman School of Public Health, Columbia University, New York, New York, United States of America; 3 Department of Community Health, National University of Rwanda School of Public Health, Kigali, Rwanda; 4 Bill & Melinda Gates Foundation, Seattle, Washington, United States of America; 5 Epidemiology and Biostatistics Program, City University of New York School of Public Health at Hunter College, New York, New York, United States of America; 6 Rwanda Biomedical Center (RBC), Ministry of Health, Kigali, Rwanda; 7 National Reference Laboratory (NRL), Kigali, Rwanda; Centers for Disease Control and Prevention, United States of America

## Abstract

**Background:**

Generalizable data are needed on the magnitude and determinants of adherence and virological suppression among patients on antiretroviral therapy (ART) in Africa.

**Methods:**

We conducted a cross-sectional survey with chart abstraction, patient interviews and site assessments in a nationally representative sample of adults on ART for 6, 12 and 18 months at 20 sites in Rwanda. Adherence was assessed using 3- and 30-day patient recall. A systematically selected sub-sample had viral load (VL) measurements. Multivariable logistic regression examined predictors of non-perfect (<100%) 30-day adherence and detectable VL (>40 copies/ml).

**Results:**

Overall, 1,417 adults were interviewed and 837 had VL measures. Ninety-four percent and 78% reported perfect adherence for the last 3 and 30 days, respectively. Eighty-three percent had undetectable VL. In adjusted models, characteristics independently associated with higher odds of non-perfect 30-day adherence were: being on ART for 18 months (vs. 6 months); younger age; reporting severe (vs. no or few) side effects in the prior 30 days; having no documentation of CD4 cell count at ART initiation (vs. having a CD4 cell count of <200 cells/µL); alcohol use; and attending sites which initiated ART services in 2003–2004 and 2005 (vs. 2006–2007); sites with ≥600 (vs. <600 patients) on ART; or sites with peer educators. Participation in an association for people living with HIV/AIDS; and receiving care at sites which regularly conduct home-visits were independently associated with lower odds of non-adherence. Higher odds of having a detectable VL were observed among patients at sites with peer educators. Being female; participating in an association for PLWHA; and using a reminder tool were independently associated with lower odds of having detectable VL.

**Conclusions:**

High levels of adherence and viral suppression were observed in the Rwandan national ART program, and associated with potentially modifiable factors.

## Introduction

In recent years, HIV care and treatment programs in sub-Saharan Africa have shifted from an emergency response with a focus on quickly initiating the sickest HIV-infected patients on antiretroviral therapy (ART) to building sustainable programs which provide lifelong treatment to very large numbers of patients across the HIV disease spectrum. Among the pillars of sustainable HIV treatment programs is the ability of patients to achieve and maintain adequate adherence to ART for life. Adherence is critical for improving the patient’s own prognosis [1], [2], minimizing development of drug resistant HIV [3], [4], and reducing the risk of HIV transmission to HIV-negative sexual partners [5], [6].

In contrast to the growing literature on levels and determinants of patient retention from nationally representative or large multi-site samples of ART patients in Africa [7]–[10], generalizable data on patient adherence to ART in sub-Saharan Africa are limited. A meta-analysis of 27 small observational studies (median sample size = 100 patients) conducted in Africa during a very early phase of ART scale-up reported adequate adherence among 77% of patients [11]. More recent single-site and small multi-site reports from service-delivery settings in the region have reported optimal adherence among 25% to 94% of patients [2], [12]–[14]. Additionally, virologic monitoring, an objective measure of adherence used regularly for patient management and program evaluation in high-income settings, is rarely conducted in Africa. A recent systematic review of 89 African studies with any virologic data conducted largely in urban settings reported undetectable viral loads among 78% of patients after six months of ART, 76% after 12 months, and 67% after 24 months [15].

Rwanda has an estimated adult national HIV prevalence of 3% (2% in men and 4% in women) [16] and is one of three low- and middle-income countries with a generalized HIV epidemic to have achieved universal access to ART [17]. By December 2010, 91,984 people were receiving ART, representing 88% of the population estimated to be in need of treatment [17]. As in many sub-Saharan African countries, the Rwandan national ART program expanded rapidly from 4 clinics in 2002 to 328 clinics by 2010 [18], [19]. An evaluation of the national program for the 2004–2005 period revealed that 92% and 84% of patients were retained six and 12 months after ART initiation, respectively [9]. Data on adherence are available from two single-site studies, both conducted in the capital city of Kigali, and suggest very high levels of perfect adherence at 12 months [20], [21]. Neither study assessed determinants of optimal adherence or included other measures of adherence. We use data from a large, nationally representative, multi-site study of the magnitude and determinants of self-reported adherence, treatment interruptions, and virological suppression among patients remaining on ART for 6, 12 and 18 months in the Rwandan national ART program.

## Methods

### Study Design, Population and Sampling

By the end of February 2007, approximately 18 months prior to study start, 113 health facilities, including 79 public facilities and 34 faith-based facilities, were providing ART, and according to the national monitoring and evaluation system, 9,693 adults were on ART: 3,628 for 6 months 3,086 for 12 months, and 2,979 for 18 months. From September 2008 to April 2009, we conducted a cross-sectional study in 20 of the 113 facilities.

Sample size calculations were based on the expected proportion of patients reporting perfect adherence 18 months after ART initiation as that proportion was expected to be lower than those at 6 and 12 months after ART initiation. Assuming an 18-month perfect adherence rate of 85%, a precision of ±5% or less, a design effect of 1.5, and a refusal rate of 25%, as well as application of a finite population correction factor and logistical considerations, a sample of 1,798 patients spread across 20 sites was considered adequate.

Study participation was restricted to adults aged ≥18 years at study enrolment who had initiated first-line ART at one of the study sites 6, 12 and 18 months [+/−2 months] prior to data collection and were still receiving treatment at their initiating site, or transferred into one of the study sites within 30 days of ART initiation and were still receiving it at that site. Patients who died, were lost to follow-up (defined according to national guidelines as no clinic visit for 90 days or more since the last documented visit), transferred to another clinic before study start were excluded, as were those who continued in care at their initiating site but had stopped ART prior to data collection.

Stratified multi-stage cluster sampling was used, with sites as clusters, and six strata according to type of facility (public and faith-based) and time on ART (6, 12 and 18 months). The first stage of sampling involved randomly selecting 14 public and 6 faith-based sites from the 113 sites providing ART services 18 months prior to study start, with the total number of each type of site determined based on the relative contribution of each of those types of sites to the total number of adults on ART in Rwanda recorded in the national monitoring and evaluation system. In the second stage of sampling, patient registers and charts at the selected sites were used to create sampling frames of all eligible patients per strata. The desired sample size per strata was determined based on the relative contribution of eligible patients in each stratum to the total number of eligible patients identified. The sample per strata was divided across sites based on the relative contribution of eligible patients at each site to the total number of patients required per strata. Potential participants were selected from the site-specific sampling frames using simple random sampling. Individual records were reviewed for each potential participant selected using random sampling to confirm that they met the study eligibility criteria. If a patient was found to be ineligible, she or he was replaced by another eligible patient from the appropriate site-specific sampling frame using a random replacement scheme.

Site staff otherwise unaffiliated with the study contacted the selected patients confirmed to meet the study eligibility criteria at home and invited them to return to the health facility to learn more about the study. For patients who presented to the clinic, study staff provided more details about the study, obtained written informed consent, and completed interviews. Every alternate participant was included in the viral load sub-sample, with blood draw occurring immediately after the interview. Participants received the equivalent of US $4.50 to cover transport to the clinic to participate in the study. Patients who did not present to the facility following the invitation by the site staff, refused to participate after presenting to the facility, or could not be located were not replaced.

### Study Procedures

#### Participant interview

Trained interviewers conducted face-to-face interviews which lasted approximately 45 minutes using a closed-ended questionnaire translated into Kinyarwanda. The questionnaire covered socio-demographic characteristics, psychosocial support, ART-related side effects, health behaviors and beliefs. Adherence was measured by patient recall of the number or percentage of doses taken during the three and 30 days prior to interview. Specifically, three-day patient recall was assessed using questions developed by the Adult AIDS Clinical Trials Group which have been previously validated in the United States [22] and used successfully in low-income settings [14], [23]–[25]. For each medication prescribed, patients were asked to indicate whether they took the required doses during each of the three days preceding the interview. These questions were preceded by a statement that many people do not take their medication perfectly all of the time in an effort to elicit accurate reporting. Thirty-day recall was assessed using an ordinal visual analogue scale modeled on a continuous numeric scale validated in the United States [26] and a categorical pictorial scale developed by ICAP staff in Mozambique [27]. Patients were presented with a line anchored with cups at 0 (empty cup) and 10 (full cup), provided with examples of what 0, 50 and 100% adherence would represent, and asked to show the percentage of doses of all ART medications they took relative to that prescribed for the 30-day period prior to the interview. In order to assess treatment interruptions, patients were also asked the number of times they had missed taking all of their medication for three or more days since starting ART.

#### Abstraction of clinical data

Interviewers used a structured tool to abstract data on ART regimens, clinic visits and CD4, weight and WHO stage assessments since enrollment into care from patient medical charts and pharmacy records.

#### Viral load assessment

For patients selected into the viral load sub-sample, blood specimens were collected by the site phlebotomist in PPT or EDTA tubes (Becton Dickinson, San Jose, CA, USA). Depending on the distance of the site from the National Reference Lab (NRL) in Kigali, samples were either transported directly to the NRL for centrifuging within four hours of being drawn, or centrifuged at the site or a nearby District Hospital within four hours and then transported to the NRL within 18 hours. Plasma levels of HIV RNA were quantified by real-time PCR using a Cobas TaqMan 48 machine (Roche Diagnostic Systems, NJ, USA) with a detection limit of 40 RNA copies/mL.

#### Site assessment

Data on facility- and program-level factors that may impact adherence at the patient level were obtained for each site. Interviewers completed a structured assessment tool via discussion with the director of the facility or the HIV clinic, or another staff member familiar with the day-to-day operations of the HIV clinic.

### Data Management and Analysis

Data were double data entered into a Questionnaire Design Software (QDS™) (NOVA Research Company, Bethesda, MD) database and analyzed using survey procedures in SAS Version 9.2 (SAS, Cary, NC) designed for complex survey data. Sampling weights accounting for the probability that a site would be selected from the 113 in the sampling frame (by type of site: public and faith-based) and the probability that a patient would be selected from the site-specific sampling frame of all patients on ART (by time on ART: 6, 12, and 18 months) were used together with finite population correction factors in all analyses to obtain nationally representative estimates. Descriptive statistics were used to describe study sites and patient characteristics by time since ART initiation. Principal components analysis considering information on dwelling conditions and household assets was used to create a poverty index which was then divided into tertiles, representing the poorest, middle and least poor respondents. An index of side effects was generated by summing scaled responses to whether each of 19 side effects were experienced and if so, their severity, in the 30 days prior to interview; the index was categorized based on the 25^th^ and 75^th^ percentile cut-offs, representing whether the respondent experienced no or few side effects, moderate side effects or severe side effects. If CD4 counts were not available from the visit at which ART was initiated, results from up to three months before or after the date of ART initiation were used.

Primary outcome measures were three-day adherence, 30-day adherence, treatment interruptions, and viral load at the time of interview. Adherence and viral load were treated both continuously and categorically using clinically relevant thresholds. Treatment interruptions were examined using rates per person-year on ART. The four measures were compared across time since ART initiation strata using descriptive statistics, as appropriate. Logistic regression analysis was used to identify patient- and site-level characteristics associated with two of those outcomes: sub-optimal 30-day adherence and detectable viral load. Sub-optimal adherence was defined as patient report of having taken less than 100% of prescribed doses in the 30 days prior to interview, and viral loads of more than 40 copies/mL were designated as detectable. Non-collinear factors significant at the α≤0.2 level in unadjusted models were introduced in multivariable models. Variables that were statistically significant at α≤0.05 and that contributed to the overall goodness of fit of the model were retained in the final models. If not independently associated with the outcome, time since ART initiation, age, sex and CD4 at ART initiation were forced into the final models regardless of their statistical significance. Characteristics associated with three-day adherence and treatment interruptions were not assessed due to insufficient variability in the data.

### Ethical Considerations

The study protocol was approved by the National AIDS Commission, the National Ethics Committee and the National Institute of Statistics in Rwanda, as well as by the Institutional Review Board at Columbia University. All participants provided written informed consent prior to interview, and when applicable, prior to blood draw for viral load assessment.

## Results

**Figure 1:**
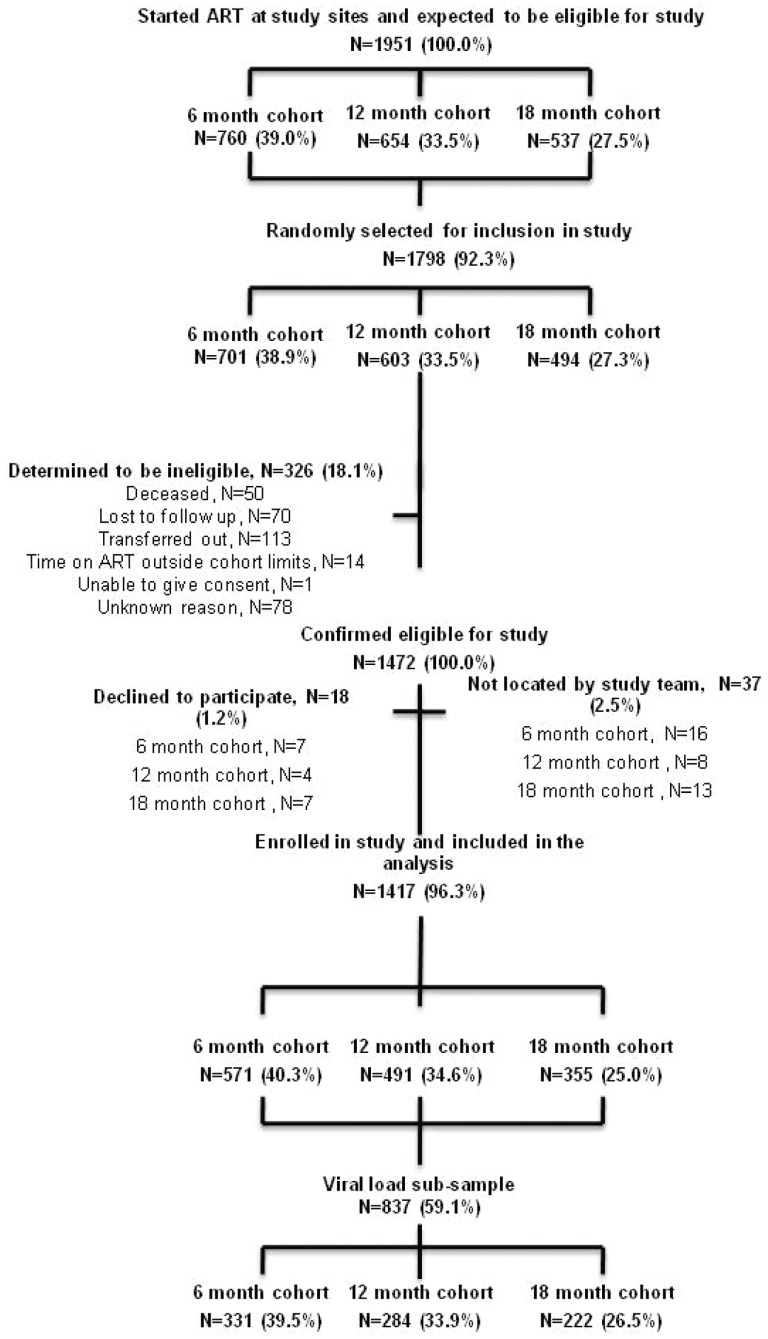
Participant selection and recruitment.

According to government data, a total of 1,951 patients were believed to have started ART 6, 12 and 18 months prior to data collection at the 20 study sites, 1,798 (92%) of whom were randomly selected for inclusion in the study. Of those selected for inclusion, 1,472 (82%) were confirmed to meet the study eligibility criteria following individual record review, and 1,417 (96%) of those patients could be located and agreed to participate, including 571 (40%), 491 (35%) and 355 (25%) who started ART 6, 12 and 18 months prior to data collection, respectively. A total of 837 (59%) of the 1,417 participants received viral load assessments, including 331 (40%) who had been on ART for 6 months, 284 (34%) on for 12 months and 222 (27%) on for 18 months (Figure 1). After applying the sampling weights, the total population was 6,996, with 2,724 (39%), 2,353 (34%) and 1,921 (27%) in the 6, 12, and 18 months on ART groups, respectively. The weighted viral load population was 4,184 (60%), with 1,598 (38%) 1,336 (32%) 1,250 (30%) in the 6, 12, and 18 months on ART groups, respectively.

### Site Characteristics

Of the 20 study sites, 14 (70%) were public sector and six (30%) were faith-based, as per the study design. Eleven (55%) were located in rural areas. There were 14 (70%) primary-level health centers and six (30%) secondary-level hospitals. Seven (35%) sites had started providing ART services in 2003 or 2004, seven (35%) in 2005 and six (30%) in 2006 or 2007. Six (30%) sites had active peer educator programs and ten (53%) conducted routine home visits for patients.

### Population Characteristics

Table 1 shows the characteristics of the population on ART for 6, 12 and 18 months in Rwanda at the time of data collection. There were few statistically significant differences in the distribution of population characteristics by time since ART initiation. The majority of the population was female (65%) and, on average was 38 years old and had completed 5 years of school. Many patients did not have information in their medical records on their WHO stage (37%) or CD4 cell count (16%) at ART initiation, with more missing information on WHO stage observed among those who started ART more recently than those who had been on ART for a longer period of time (50%, 33%, and 24% missing for 6, 12, 18 month cohorts, respectively). Among those with information available on their disease stage at ART initiation, there was variation by time since ART initiation, with patients on ART for a longer period of time starting ART in a more compromised health stage (median CD4 counts of 258, 209, and 192 cells/µl for 6, 12, 18 month cohorts, respectively). The majority (89%) of patients were on Nevirapine-based regimens and on average took 1.6 pills per day. About one-quarter (26%) reported severe side effects in the 30 days before interview. Nearly (94%) all believed ART was “very effective”, more than half (57%) used at least one ART reminder tool, with alarm clocks used most often by 22% of the population, and 53% participated regularly in PLWHA support groups. Thirteen percent reported consuming alcohol on at least one day in the week prior to data collection.

**Table 1:** Patient characteristics by time on ART.

			Total		6 months		12 months		18 months		
			N	Weighted %	N	Weighted %	N	Weighted %	N	Weighted %	p-value
			1417	100.0	571	40.0	491	35.0	355	25.0	
***Sociodemographic Factors***											
**Sex**											
Male			499	35.0	201	35.1	170	35.2	128	34.5	0.982
Female			913	65.0	369	64.9	319	64.8	225	65.5	
Missing			5		1		2		2		
**Age**											
Mean years, SE			38.1	0.3	38.2	0.4	38.4	0.4	37.4	0.6	0.522
Age groups											
18–24 years			101	6.3	41	6.0	35	6.4	25	6.6	0.960
25–34 years			480	32.7	191	32.7	161	30.4	128	35.6	0.500
35–44 years			531	36.9	208	35.0	182	37.5	141	38.7	0.749
≥45 years			305	24.1	131	26.3	113	25.7	61	19.1	0.146
Missing			0		0		0		0		
**Education**											
Mean years, SE			5.0	0.2	4.9	0.4	4.7	0.3	5.5	0.3	0.291
Education levels											
None			321	26.4	129	25.3	114	29.5	78	24.1	0.577
Some			1096	73.6	442	74.7	377	70.5	277	75.9	
Missing			0		0		0		0		
**Marital status**											
Married/living with partner			771	53.5	307	52.5	275	54.7	189	53.7	0.824
Separated/divorced/Widowed/not living with partner			515	38.2	206	39.2	178	37.9	131	37.3	
Never married			125	8.3	57	8.3	37	7.4	31	9.2	
Missing			6		1		1		4		
**Number of household members**											
Mean number, SE			5	0.0	5	0.1	5	0.1	5	0.1	0.841
Missing			7		3		1		3		
**Household poverty index**											
Poorest			490	42.6	198	41.0	180	47.1	112	39.3	0.880
Less poor			455	33.4	184	35.6	152	30.2	119	34.1	
Least poor			472	24.0	189	23.4	159	22.7	124	26.6	
Missing			0		0		0		0		
***Treatment-related Factors***											
**WHO stage at ART initiation**											
I & II			523	38.1	184	32.8	182	41.6	157	41.2	0.001
III & IV			373	24.9	100	16.9	150	25.8	123	35.0	
Missing			521	37.0	287	50.3	159	32.6	75	23.8	
**CD4 count (cells/µL) at ART initiation**											
Median, IQR			221.9	149.9–293.7	258.0	184.1–309.8	209.2	139.6–287.8	191.8	130.8–270.3	<0.001
<200			560	35.0	156	25.0	224	40.0	180	43.2	0.004
≥200			651	49.1	318	57.8	205	46.4	128	39.9	
Missing			206	15.9	97	17.1	62	13.6	47	16.9	
**Current ART regimen**											
Nevirapine-based			1220	89.1	507	91.7	411	85.7	302	89.4	0.127
Efavirenz-based			167	10.3	53	7.5	66	13.6	48	10.1	
Other			9	0.7	3	0.7	3	0.7	3	0.4	
Missing			21		8		11		2		
**Number of daily pills**											
Median, IQR			1.6	1.3–1.9	1.6	1.3–1.8	2.0	2.0–2.0	1.6	1.3–1.8	0.043
Missing			98		34		32		32		
**Side effects index**											
No or few side effects			371	26.1	154	27.1	114	24.0	103	27.4	0.789
Moderate side effects			690	48.1	276	45.7	247	51.4	167	47.6	
Severe side effects			350	25.7	139	27.2	127	24.6	84	25.0	
Missing			6		2		3		1		
***Psychosocial and Behavioral Factors***											
**Perception of ART effectiveness in keeping respondent healthy**											
Very effective			1334	94.0	530	91.4	469	96.6	335	94.6	0.002
Somewhat effective/not effective at all			83	6.0	41	8.6	22	3.4	17	5.4	
Missing			0		0		0		0		
**Participates regularly in PLWHA meeting**			749	52.5	246	46.7	234	52.7	181	60.6	0.181
Missing			0		0		0		0		
**Frequency of alcohol consumption**											
A lot (4–7 days)			7	1.3	4	1.0	1	0.2	2	3.2	0.115
Some (1–3 days)			49	11.7	17	7.9	22	16.3	10	12.0	
Never			290	87.0	118	91.1	106	83.5	66	84.8	
Missing			6		3		2		1		
**Reminder tools to take ART**											
No tools			522	42.6	202	44.2	186	42.4	134	40.5	0.846
Cell phone			243	13.1	99	13.4	78	12.8	66	13.0	0.991
Alarm clock			322	21.9	141	22.6	107	21.6	74	21.4	0.962
Paper diary			32	2.0	12	1.7	9	1.6	11	2.9	0.606
Radio			271	17.8	115	18.2	92	16.1	64	19.2	0.761
Other			85	5.4	33	4.4	31	6.2	21	5.9	0.675
Use any tool			894	57.4	369	55.8	304	57.6	221	59.5	0.846
Missing			1		1		1		1		

### Levels of Self-reported Adherence, Treatment Interruptions and Viral Suppression

**Figure 2:**
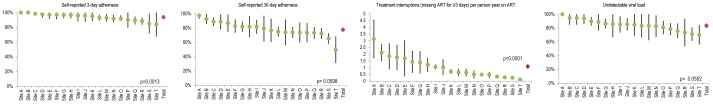
Proportion (and 95% CI) of patients reporting 100% adherence during 3 days or 30 days preceding interview, rate of treatment interruptions (missing ART for ≥3 days) per person-year on ART (95% CI), and proportion (95% CI) of patients with undetectable viral load by site.

As shown in Table 2, 94% of the population reported perfect three-day adherence. Perfect adherence in the 30 days preceding the interview was reported by 78% of the population. An additional 11% took 90–99% of all their pills in the 30 days prior to data collection, 7% took 80%–89, and 4% took less than 80%. The rate of treatment interruptions was one 1.1 per person-year on ART. Eighty-three percent of the population had an undetectable viral load, 9% had a viral load of 41–500 copies/mL and 8% had a viral load of >500 copies/mL. While none of these measures varied by time on ART, significant differences were observed by site (Figure 2), with perfect 3-day adherence ranging from 84% to 100% (p = 0.0013) across sites, perfect 30-day adherence ranging from 50% to 98% (p = 0.0598), treatment interruptions ranging from 0.1 to 3.1 per person-year on ART (p<0.0001), and viral suppression ranging from 70% to 100% (p = 0.0552).

**Table 2:** Adherence by time on ART.

			Total		6 months		12 months		18 months	
		N	Weighted %	N	Weighted %	N	Weighted %	N	Weighted %	p-value
		1417	100.0	571	40.0	491	35.0	355	25.0	
**Patient 3-day recall**										
100% adherent (95% CI)		1416	93.8 (92.8–94.8)	553	94.1 (92.8–95.4)	456	95.0 (93.9–96.5)	331	91.7 (88.9–94.4)	0.238
90–99% adherent		57	3.1	22	2.3	22	3.2	13	4.2	0.533
80–89% adherent		21	1.3	6	0.6	9	1.3	6	2.1	0.139
<80% adherent		19	1.9	10	3.0	4	0.5	5	2.0	0.083
Median, IQR		94.7	92.0–97.3	94.7	92.0–97.3	94.7	92.1–97.4	94.5	91.8–97.3	0.265
Missing		1		0		1		0		
**Patient 30-day recall**										
100% adherent (95% CI)		1086	77.6 (74.9–80.2)	422	78.5 (74.1–82.9)	376	79.4 (75.2–83.6)	268	74.0 (68.6–79.4)	0.808
90–99% adherent		163	11.3	64	10.8	54	9.2	45	14.6	0.265
80–89% adherent		103	6.7	39	6.1	34	7.6	30	6.4	0.854
<80% adherent		56	4.1	25	4.5	22	3.3	9	4.4	0.859
Median, IQR		93.6	90.3–96.8	93.6	90.4–96.8	93.7	90.6–96.9	93.2	89.3–96.6	0.938
Missing		9		1		5		3		
**Viral load (copies/mL)**										
Number with viral load		837	59.8	331	58.7	284	56.8	222	65.1	
Undetectable/≤40 (95% CI)		693	83.3 (81.7–90.7)	275	82.4 (79.7–85.0)	232	81.8 (78.5–85.1)	186	86.2 (81.7–90.7)	0.548
41–500		81	8.9	35	10.8	28	9.4	18	6.1	0.558
>500		63	7.7	21	6.9	24	8.8	18	7.7	0.558
Median VL among detectable VL, IQR		316.7	86.7–5426.8	248.2	77.0–1318.4	274.1	84.6–6136.6	1498.8	113.1–55656.0	0.300
**Rate of treatment interruptions (missing ART for ≥3 days) per person-year on ART**										
Rate, SE		1.1	0.1	1.2	0.2	1.1	0.2	0.8	0.2	0.147
Missing		6		2		1		3		

### Characteristics Associated with Non-perfect Adherence and Detectable Viral Load

Table 3 shows characteristics associated with reporting non-perfect 30-day adherence and having a detectable viral load in unadjusted and adjusted analyses. In adjusted models controlling for sex, characteristics independently associated with higher odds of non-perfect 30-day adherence were being on ART for 18 months vs. 6 months (AOR = 1.75, 95% CI [1.18–2.60]), being aged 18–24, 25–34 and 35–44 vs. ≥44 (AOR = 2.00, 95% CI [1.05–3.83]; AOR = 2.51, 95% CI [1.50–4.20]; AOR = 2.06, 95% CI [1.28–3.30], respectively), reporting severe vs. no or few side effects in the prior 30 days (AOR = 2.18, 95% CI [1.47–3.24]); having no documentation of CD4 cell count at ART initiation vs. having a CD4 cell count of <200 cells/µL (AOR = 1.65, 95% CI [1.03–2.64]); alcohol use (AOR = 1.55, 95% CI [1.01–2.38]); and attending sites which initiated ART services in 2003–2004 and 2005 vs. 2006–2007 (AOR = 1.82, 95% CI [1.07–3.09]; AOR = 2.11, 95% CI [1.23–3.62], respectively); sites with ≥600 vs. <600 patients currently on ART (AOR = 1.63, 95% CI [1.20–2.23]) or those with peer educators (AOR = 2.94, 95% CI [1.87–4.64]). Participation in an association for people living with HIV/AIDS (AOR = 0.60, 95% CI [0.42–0.87]); and receiving care at sites which regularly conduct home-visits (AOR = 0.60, 95% CI [0.42–0.87]) were independently associated a lower risk of non-adherence.

**Table 3:** Bivariate and multivariate association of patient-and site level predictors and self-reported 30-day non-adherence (<100% adherent) and detectable viral load (>40 copies/mL).

		Non-adherence				Detectable viral load			
		Unadjusted		Adjusted		Unadjusted		Adjusted	
		N = 1408		N = 1395		N = 835		N = 828	
		20 sites		20 sites		20 sites		20 sites	
		OR	95% CI	AOR	95% CI	OR	95% CI	AOR	95% CI
PATIENT LEVEL FACTORS	**Duration on ART (ref = 6 months)**								
	12 months	0.95	0.57–1.57	1.31	0.89–1.94	1.04	0.79–1.38	1.07	0.73–1.55
	18 months	1.28	0.75–2.20	1.75	1.18–2.60	0.75	0.50–1.14	0.79	0.47–1.33
	***Sociodemographic Factors***								
	**Age (ref = ≥45 yrs)**								
	18–24 yrs	1.87	1.31–2.67	2.00	1.05–3.83	2.12	1.08–4.14	2.56	0.98–6.74
	25–34 yrs	2.79	2.16–3.60	2.51	1.50–4.20	1.41	0.83–2.40	1.74	0.75–4.06
	35–44 yrs	1.94	1.53–2.46	2.06	1.28–3.30	0.96	0.60–1.55	1.11	0.52–2.39
	**Sex (ref = Male)**								
	Female	1.04	0.82–1.33	0.90	0.57–1.43	0.68	0.48–0.97	0.52	0.29–0.94
	**Education (ref = no education)**								
	Some education	1.52	1.00–2.32			1.56	1.07–2.28		
	**Current marital status (ref = Married/living together)**								
	Other	0.75	0.57–0.99						
	**Total number of household members (ref = ≤4)**								
	5–6	0.76	0.59–0.99						
	≥7	1.05	0.76–1.46						
	**Poverty index (ref = most poor)**								
	Middle	1.42	1.07–1,89						
	Least poor	1.63	1.30–2.05						
	***Treatment-related Factors***								
	**Side effects in past 30 days (ref = none/few)**								
	Moderate	1.36	1.01–1.84	1.45	0.91–2.31				
	Severe	2.01	1.56–2.59	2.18	1.47–3.24				
	**CD4 count at ART initiation (ref = <200)**								
	≥200	1.05	0.76–1.44	1.31	0.86–1.98	0.87	0.65–1.81	0.85	0.50–1.44
	Missing	1.22	0.89–1.67	1.65	1.03–2.64	1.15	0.77–1.70	1.13	0.58–2.20
	***Psychosocial and Behavioral Factors***								
	**Perception of ART effectiveness (ref = effective)**								
	Not effective	2.14	1.42–3.2						
	**Participates in PLWHA association (ref = no)**								
	Yes	0.61	0.52–0.73	0.66	0.48–0.90	0.39	0.28–0.56	0.39	0.24–0.65
	**Frequency of alcohol consumption (ref = none)**								
	Some or a lot	1.65	1.27–2.16	1.55	1.01–2.38				
	**Uses any reminder tool to take ART (ref = no)**								
	Yes					0.59	0.45–0.78	0.45	0.29–0.70
SITE LEVEL FACTORS	**Facility ownership (ref = Faith-based)**								
	Public					0.72	0.57–0.93		
	**Site location (ref = rural)**								
	Urban	2.10	1.61–2.73			1.46	1.12–1.90		
	**Site type (ref = health centre)**								
	Hospital					1.41	1.06–1.88		
	**Year ART services initiated (ref = 2006–2007)**								
	2003–2004	1.39	1.05–1.83	1.82	1.07–3.09				
	2005	1.68	1.17–2.41	2.11	1.23–3.62				
	**Site ART enrollment (ref = <600)**								
	≥600 patients	1.40	1.11–1.77	1.63	1.20–2.23				
	**Peer educator program (ref = no)**								
	Yes	1.79	1.33–2.40	2.94	1.87–4.64	1.77	1.36–2.30	2.01	1.40–2.88
	**Home support visits for PLWHA (ref = no)**								
	Yes	0.60	0.46–0.79	0.60	0.42–0.87				

* Note: The variables cohort, CD4 at initiation, sex and age have been forced to remain in the model.

In adjusted models controlling for duration on ART, age and CD4 count at ART initiation, higher odds of having a detectable VL were observed among patients at sites with peer educators (AOR = 2.01, 95% CI [1.40–2.88[). Being female (AOR = 0.52, 95% CI [0.29–0.94]); participating in an association for people living with HIV/AIDS (AOR = 0.39, 95% CI [0.24–0.65]); and using a reminder tool (AOR = 0.45, 95% CI [0.29–0.70]) were negatively associated with having a viral load of more than 40 copies/mL.

## Discussion

As the first nationally representative study on adherence, treatment interruptions and viral suppression among patients on ART in sub-Saharan Africa, this study provides important insights on program outcomes previously not sufficiently described in the context of rapid scale-up of HIV services. Additionally, as Rwanda is one of the small handful of countries in sub-Saharan Africa that has achieved universal treatment coverage, we were able to assess whether program quality can be maintained as countries reach that goal. Reassuringly, very high program quality was observed. Overall, across the population on ART for 6, 12 and 18 months in Rwanda, 94% reported perfect three-day adherence and 78% reported perfect 30-day adherence. This finding is consistent with that reported in smaller studies in East Africa [1], [14], but lower than that reported from single-site studies in Kigali [11], [20], [21]. Additionally, reports of treatment interruptions of three days or more were rare, resulting in a low rate of interruptions of one per person-year on ART. Patients in our study also demonstrated a high degree of viral suppression with 83% having undetectable viral loads, considerably higher than that observed in a large sample from a South African program setting where 62% of adult patients had viral load ≤400 copies/mL at 12 months [2].

A number of patient characteristics were associated with higher odds of non-adherence, including longer time on ART, younger age, experiencing severe side effects, not having a CD4 count at ART initiation, and alcohol use. Patients who had been on ART for 18 months had 75% higher odds of non-adherence compared to those who had been on ART for 6 months. This trend was also observed among adults in a South Africa [2] and suggests that patients who have been on ART longer should be targeted for adherence support interventions. The association between younger age and increased risk for non-adherence has also been demonstrated by other studies [2], [25] implying a need for focused interventions to support adherence among younger adults. Side effects are a recognised barrier to ART adherence [28] and should be effectively managed for better adherence. Encouragingly, a large proportion of patients (84%) in a task shifting study in Rwanda had side effects regularly assessed [29], and this practice should be encouraged at all clinics. The association between lack of a CD4 at ART initiation and higher odds of non-adherence may mean that patients fail to grasp the severity of their illness and the necessity of treatment. Understanding one’s HIV disease and medications for managing may support patient adherence [30]–[32]. The detrimental impact of alcohol consumption on ART adherence has been well documented in other studies [28], [33]–[36]. Assessment for significant alcohol use should be integrated into routine adherence counselling and prevention with positives interventions [37].

Several patient characteristics were also independently associated with lower odds of non-adherence and having a detectable viral load, and suggest avenues for intervention among patients experiencing adherence problems. Indeed, participation in a PLWHA association, which we found to be associated with decreased risk of non-adherence and having a detectable viral load, may encourage positive behaviour among patients on ART. Additionally, while use of reminder tools was not associated with self-reported adherence, their use was significantly associated with low odds of having a detectable viral load, providing further evidence that they promote adherence [38]–[40]. Women were also less likely to have a detectable viral load, consistent with a recent study from Uganda, but no gender differential was observed in reporting of non-adherence. This is consistent with data from a smaller study in Uganda [41] and suggests either a true sex difference in response to ART or misreporting of adherence among men.

Significant variability by site was observed in all study outcomes, suggesting opportunities exist for site-level interventions to optimize outcomes. Patients attending high volume sites had higher odds of non-adherence probably due to insufficient staff time to focus on this aspect of patient management. Patients attending sites that had started implementation of ART services earlier in scale-up also had higher odds of non-adherence than those attending less mature sites. More research is needed to understand this finding, including assessing the contribution of health worker fatigue. Such sites might benefit from implementing supportive home visits, which were associated with reduced odds of non-adherence in our study. In contrast, we found that patients attending sites with peer educator programs had higher odds of non-adherence and of having a detectable viral load. While some studies suggest that peer support is associated with better adherence, particularly if it includes implementation of directly observed therapy [42], [43], a recent large cluster randomised study from Uganda found no impact of peer educators on adherence and viral suppression up to 96 weeks [44]. As the cross-sectional nature of our study precludes us from ruling out reverse causality, such that sites which experienced poor adherence and treatment response preferentially implemented peer educator programs, further studies are needed to understand the role of peer educator programs in this setting.

Our study has several important strengths. To our knowledge, it is the first nationally representative assessment of adherence, viral suppression, and treatment interruptions among patients on ART in Africa and possibly worldwide. Having both patient-level and site-level data allowed us to explore a wide range of correlates of optimal adherence and viral suppression. A few limitations should be noted, however. While the objective of our study was to assess outcomes among patients who remained on treatment for 6, 12 and 18 months, it is likely that those who were not retained had worse adherence and more treatment interruptions, and thus were less likely to achieve viral suppression. Similarly, while non-participation was extremely rare and restricted to 55 (3.7%) of patients confirmed to be eligible for the study, these patients likely had worse outcomes than study participants; indeed, assuming all such patients had non-perfect adherence and detectable viral loads, the overall weighted proportion of participants with perfect 3-day and 30-day adherence, and undetectable viral load would be 90%, 74%, and 78%, respectively. Additionally, lack of variation in some site-level variables precluded their inclusion in multivariable models. Finally, due to financial constraints, we were unable to perform viral load assessments for all patients which limited our power to detect significant associations when modelling predictors of detectable viral load.

In conclusion, high levels of self-reported adherence and virological suppression, and low rates of treatment interruption were observed among a nationally representative sample of patients on ART for 6, 12 and 18 months in the Rwandan national program. Our results suggest that strategies to maximize adherence in these settings should include reminder tools, alcohol screening and treatment, participation in PLWHA associations and supportive home visits.
